# Assessment of Extra-Cortical Bone Bridge Interface in Cemented Mega Endoprosthesis for Limb Salvage Surgery

**DOI:** 10.5704/MOJ.2103.014

**Published:** 2021-03

**Authors:** K Murugan, WI Faisham, W Zulmi

**Affiliations:** Department of Orthopaedics, Universiti Sains Malaysia, Kubang Kerian, Malaysia

**Keywords:** extra-cortical bone bridge (EBBI), endoprosthesis, PACS

## Abstract

**Introduction:**

Mega endoprosthesis replacement for resection of primary malignant bone tumour requires immediate and long-term stability, particularly in the young and active patient. Extracortical bone bridge interface (EBBI) is a technique whereby autograft is wrapped around the interface junction of bone and porous-coated implant to induce and enhance bone formation for biological incorporation. This procedure increases the mean torsional stiffness and the mean maximum torque, which eventually improves the implant's long-term survival.

**Material and methods:**

The extracortical bone bridge interface's radiological parameter was evaluated at the prosthesis bone junction two years after surgery utilising a picture archiving and communication system (PACS). The radiograph's anteroposterior and lateral view was analysed for both thickness and length in all four cortices. The analysis was done in SPSS Version 24 using One-Way ANOVA and independent T-Test. Results were presented as mean and standard deviation and considered significant when the p-value was < 0.05.

**Results:**

The mean average thickness was 2.2293mm (SD 1.829), and the mean average length was 31.95% (SD 24.55). We observed that the thickness and length of EBBI were superior in the young patient or patients with giant cell tumour that did not receive chemotherapy, compared to patients treated for osteosarcoma. The distal femur also had better EBBI compared to the proximal tibia. However, the final multivariable statistical analysis showed no significant difference in all variables. EBBI thickness was significantly and positively correlated with EBBI Length (p<0.001). We conclude that, for each 1mm increase in EBBI thickness, the length will increase by 0.06% on average. About 17.2% of patients out of the 29 showed no radiological evidence of EBBI.

**Conclusion:**

From our study, there were no factors that significantly contributed to the formation and incorporation of EBBI

## Introduction

Mega endoprosthesis replacement for resection of primary malignant bone tumour requires immediate and long-term stability, particularly in the young and active patient. There is still controversy in the use of cementless or cemented prosthesis in terms of surgical advantages and long-term results. Cemented prosthesis fixation is a base filler that evenly transfers the load from the stem to the bone and absorbs loading. Due to the intramedullary smoothness of diaphysis bone, rotational and bending forces are easily transmitted and lead to cement crack and early failure. Extracortical bone bridge interface is (EBBI) a technique where autograft is wrapped around the interface junction of bone and porous-coated implant to induce and enhance bone formation for biological incorporation^1^. This procedure increases the mean torsional stiffness and the mean maximum torque, which eventually improves the long-term survival of the implant^2^.

Evaluation of EBBI in terms of thickness and length around the prosthesis is predicted to prevent failure, and predisposition essential to improve the thickness. Therefore, our study is aimed towards evaluating the factors that predict the radiological outcome of EBBI in our patients.

## Material and Method

**Fig. 1:**
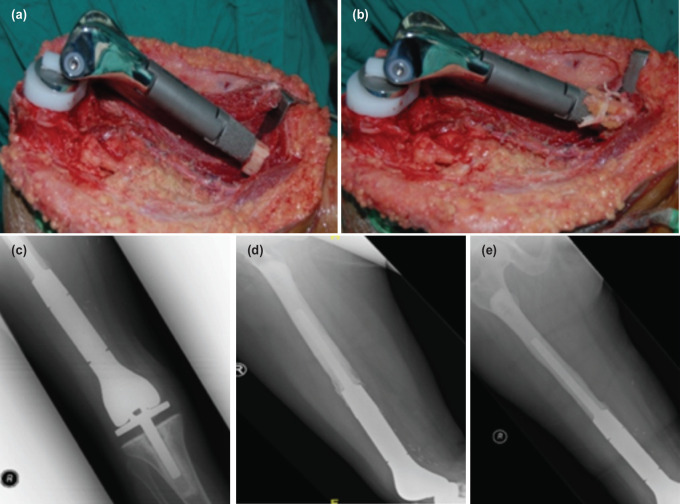
A case of distal femur endoprosthesis; minimal formation of EBBI around prosthesis junction and showed stem loosening at 15 years after surgery (not in the study as none of cases in this study had early loosening. (a) A case of distal femur osteosarcoma; wide resection reconstructed with endoprosthesis. (b) Multiple bone sleeve at bone-implant junction for formation of EBBI. (c) Antero-posterior radiograph of knee with distal femur endoprosthesis. (d) Lateral radiograph of knee with distal femur endoprosthesis (at 15 years). (e) Antero-posteior radiograph of knee with distal femur endoprosthesis (at 15 years).

**Fig. 2:**
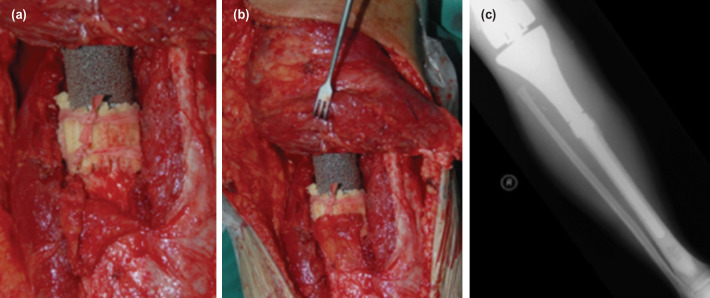
(a,b,c) Giant cell tumour of proximal tibia with EBBI and periosteal flap at 2 years, patient was follow-up with no loosening of implant at 14 years after surgery.

The radiological parameter of the extracortical bone bridge interface was evaluated at the prosthesis bone junction two years after surgery, once the junction was stabilised with local recurrence and infection are excluded. This study was a cross-sectional evaluation of 29 orthopaedic oncology patients (19 males, ten females, mean age 25 (SD 9.74), range of age between 15 and 49. Eighteen patients had osteosarcoma, and 11 patients had stage III giant cell tumours. A total of 17 distal femur and 12 proximal tibia endoprosthesis placements were carried out. Kinematic rotating-hinge prostheses were used in all patients [Stryker Howmedica Osteonic, Inc, Rutherford NJ]. The EBBI was created by utilising multiple bone sleeves measuring about 2-5mm, prepared with a vertical length of about 5cm. It was then arranged in an overlapping manner around the bone-prosthesis junction and anchored with absorbable suture. The method is illustrated in Fig. 1 and Fig. 2.

**Fig. 3:**
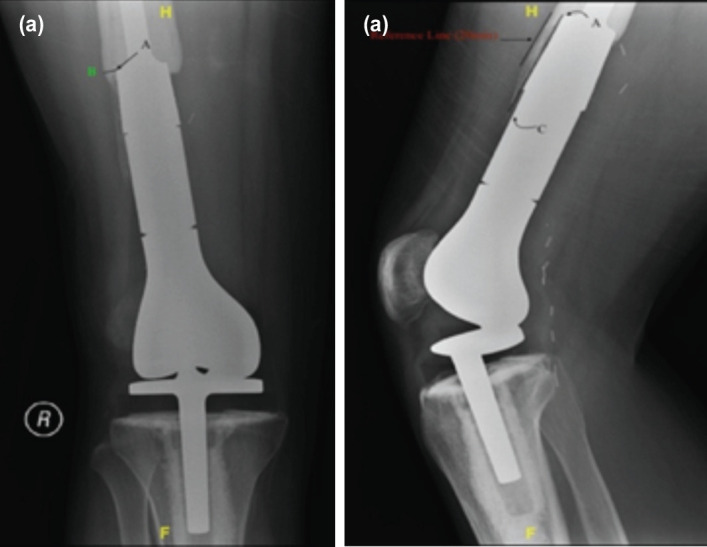
Measurement of thickness and length of extracortical bone bridge interface. (a) Antero-posterior radiograph of Knee joint shows measurement of EBBI thickness (Point A to Point B). (b) Lateral radiograph of Knee joint shows measurement of EBBI Length (Point A to Point C).

The anteroposterior and lateral view of the radiographs were used to evaluate the thickness and length of EBBI as defined. These radiographs were retrieved from the online radiological system, picture archiving, and communication system (PACS), provided by GE healthcare and accessed via https://pacszfp.usm.my/zfp. We could get reliable and accurate measurements via a soft-copy PACS system compared to hard-copy radiograph3-4. The thickness of EBBI was measured at the bone-implant junction marked as A in Fig. 3a. The thickness was measured perpendicular to the implant's long axis, so the measurement would be from point A to point B. Point B was varied according to the EBBI thickness. This measurement was done at the medial, lateral, anterior, and posterior cortex and recorded in millimetre.

The same radiograph images were used concurrently to measure the length of EBBI. For length, point A became a starting point, and measurement was done parallel to the implant's long axis up to point C, as illustrated in Fig. 3b. In contrast to thickness measurement, the length was measured as a percentage of coverage in each cortex. For instance, as shown in Fig. 3b, the EBBI length was measured 10mm, and based on our reference of 20mm, this would be reflected as 50% coverage at anterior cortex. The same technique was implied for the rest of the cortex. The maximum percentage would be 100% for each cortex. The value for the thickness and length of EBBI from each cortex was summed up and divided by four to get an average thickness and average length. This value of average thickness and length subsequently correlated with independent parameters, which were age, sex, pathology, site of pathology, and chemotherapy treatment. In this study, the average thickness and length stood as dependent parameters.

The sample size was calculated using sample size calculator v2.0 (Excel format). No literature described the measurement of length and thickness, and available literature for sample size calculation was derived from a study by Chao EY1. The sample size for comparison of mean was 38; however, due to significant dropout (23%), only 29 samples were included. Mann-Whitney U test was used for the statistical analysis in this study, and our categorical data were presented in frequency and percentage. In contrast, numerical data was presented in median and interquartile (IQR) due to the non-normal distribution of data and small sample size. We applied the Mann-Whitney U test to determine the differences in EBBI thickness and length on different factors. Besides, we also analysed the relationship between EBBI thickness and length using a simple linear regression test. All assumptions for each statistical test were checked and met. The statistical analysis was conducted using SPSS software version 24. Differences were considered significant when the p-value was <0.05. The results are presented in Table 1 as the median and p-value. Median was chosen over mean as the data was not normally distributed and skewed.

**Table I:** Table showing the variables that affect the AOFAS score

Variables		EBBI				Statistical Analysis (p-value) Mann-Whitney U Test	
		Present	Absent	Median Thickness (mm)	Median Length (%)	Thickness	Length
Age	<30	19	5	1.94	36.25	0.66	0.95
	30 above	5		2.08	32.63		
Sex	Male	16	3	2.70	38.25	0.18	0.25
	Female	8	2	1.55	24.75		
Pathology	OS	13	5	1.50	36.25	0.06	0.59
	GCT	11		2.70	36.63		
Site	PT	10	2	1.55	18.19	0.15	0.23
	DF	14	3	2.70	40.00		
Chemotherapy	Yes	13	5	1.50	36.25	0.06	0.59

## Results

All consecutive proximal tibial and distal femur endoprosthesis cases from January 2010 to December 2018 were included in his study. The extracortical bone bridge interface was measured within 24-32 months following surgery in all 29 patients with a mean age of 25 years. The average thickness of EBBI measured was 2.00mm (2.3) (ranged in between 0.0 to 6.23); meanwhile, the average length was 35.3% (40.56) (ranged in between 0 to 75) of total EBBI coverage. None of the cases in this study had early loosening with a follow-up period of a mean of 46 months (range 24 months to 135 months). Younger patients had a median thickness of 1.94mm and length of 36.253% compared to patients above 30 years old with a thickness of 2.08 and a length of 32.63%, which was not statistically significant. Osteosarcoma patients who received chemotherapy had 1.50 /36.25 thickness and length compare to GCT 2.7/36.63. The difference was minimal and was not statistically significant.

Eighteen patients with osteosarcoma, which accounted for 62.1% of the sample, showed a median thickness of 1.5mm compared to patients with giant cell tumours with a mean thickness of 2.70mm. It was statistically not significant, with a p-value of 0.06. The median length of EBBI of both osteosarcoma and giant cell tumour group of patients were also not statistically significant. The distal femur involvement accounted for 65.5% of the sample, with a median length of 40%. The median thickness was measured at 2.38mm for the distal femur and 1.55mm for the proximal tibia. The median thickness and length were not significant between these two groups, with a p-value of 0.15 and 0.23, respectively. The analysis showed that all osteosarcoma groups received chemotherapy, while the giant cell tumour patients did not receive chemotherapy. The median thickness of EBBI was 1.50mm in the chemotherapy group, and the median length was 36.25 %. These values were not significantly different from the non-chemotherapy group, 2.7mm, and 36.63%, respectively.

Nineteen male patients with 11 of them diagnosed with osteosarcoma had a median thickness of 2.70mm, while the female patients had a median thickness of 1.55mm. The value was statistically not significant with a p-value of 0.18, and the mean length of the EBBI was not statistically significant. The mean years of difference between the 1st post-operative radiograph and the available radiograph after 24 months of post-op were 3.8 years. A patient had a radiograph available after 10 years, and it showed the longevity of the implant. He had an average thickness of 2.08mm and an average length of 32.63%. Most patients fell into a group with less than five years of difference between the radiograph images (Fig. 3).

**Fig. 4:**
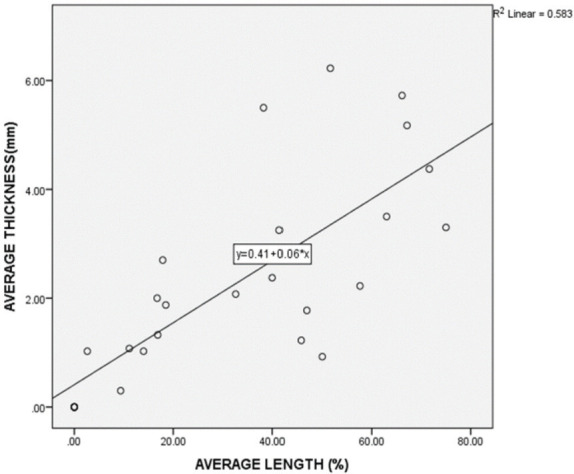
Correlation of EBBI Thickness and EBBI Length. EBBI Thickness is significantly and positively correlate with EBBI Length (p<0.001) and it also explains about 58.3% of variability (r2 = 0.583). In this study, for each 1mm increase in EBBI thickness, on average, the length will increase by 0.06%.

EBBI Thickness was significantly and positively correlated with EBBI Length (p<0.001), and it also explained about 58.3% of the variability (r2 = 0.583). In this study, for each 1mm increase in EBBI thickness, on average, the length would increase by 0.06% (Fig. 4).

Out of 29 patients, five patients (17.2%) showed no radiological evidence of EBBI incorporation during their follow-up. All these patients fell into the age group of fewer than 40 years old and were diagnosed with osteosarcoma of the proximal tibia and distal femur.

## Discussion

Patients diagnosed with sarcoma are classically treated with an amputation. With the advances in medical imaging and chemotherapy regime, limb salvage surgery can be carried out with adequate tumour clearance. It gives an overall good outcome while preserving the functional ability of the individual^5^. Amputees may be more prone to feel unattractive, report difficulties finding a life partner, and may restrict their social activities to some degree. In most centres, the endoprosthesis is being utilised for limb salvage surgery, and the standard methods are between allograft, surgical resection, and reconstruction or allograft-prosthetic composite. The choice of this endoprosthesis surgery will lead to an outcome that allows early mobilisation anda near-normal functional ability, thus preventing adverse effects such as post-operative immobilisation.

In general, endoprosthesis integration with bone could be cementless or cemented. A cemented endoprosthesis is designed with a smooth surface over the implant that integrates with the bone through bone cement. Bone cement is used together with the implant to improve bone prosthesis interlock. The micro-interlock with bone would reduce the risk of junctional loosening. Even though the micro-interlock provides additional stability, cemented implants are susceptible to fatigue failure of cement mantel in the long term due to cyclical loading and stress. Meanwhile, cementless implants provide stability by the press-fit principle^6^. New designs of cementless implants have been incorporated with hydroxyapatite porous coating to enhance longevity by increasing ingrowth within bone formation^7^.

Long-term survival of prosthesis is an essential factor in determining the outcome of limb salvage surgery. The stress, particularly impaction and rotation stress generated between bone-endoprosthesis junction, will produce aseptic loosening, failure of the construct, dislocation of the prosthetic joint, dissociation of modular components, and infection^6^. When comparing the survivorship outcome due to aseptic versus infected implant that is cemented or cementless, studies have mentioned that cemented prosthesis is highly susceptible to aseptic loosening compared to the cementless prosthesis. Still, usage of cementless stems appears to last longer in the event of infection of prosthesis^6^. Aseptic loosening remains the main factor for implant failure despite the advancement of technology; thus, few options have been established to minimise such failure. These options include Extra-Cortical Bone Bridge Interface (EBBI) in cemented stem, junctional trabecular metal, a cementless component with press-fit fixation, porous-coated implant as well as associated dynamic compression devices. The EBBI comes in handy as a solution in minimising the early failure of the endoprosthesis. It mimics the extra cortical bridging callus in the fracture union. At the initial phase, cancellous bone formed along the junction of bone and implant; eventually, it progresses into the cortical bony bridging. It has been thought to provide absolute stability for the implant externally. It allows the effective transfer of prosthesis-bone stresses, avoids stress shielding resorption, and minimises stem cement stress, thus avoid stem fracture and loosening. The EBBI also acts as a barrier by producing a cortical sleeve, which prevents bone debris entry into the bone-prosthesis junction, which may cause osteolysis. These factors enable the use of cemented prosthesis^7,8,9^. Biomechanically, the formation of EBBI increases means torsional stiffness and the mean maximum torque at failure^7^. A biomechanical study done by Fukuroku *et al* noted that augmented formation of EBBI has a significant increase in torsional stiffness and maximum torque at the failure by 2.3-fold than the control group^9^. Hydroxyapatite collars at the prosthesis bone junction have also been shown to promote bony ingrowth and decrease the rates of aseptic loosening by stabilising the stem and cement mantle, preventing the tracking of osteolytic wear debris down the canal^7^. The use of synthetic bone grafting, allograft on-lay technique, and osteogenic protein has been shown to enhance the biological fixation of bone and segmental prosthesis and replaces the need for using autogenous bone graft in the procedure. Our technique of using a remnant of non-affected side bone during endoprosthesis preparation for bone grafting precludes this procedure's necessity.

Chao *et al*; in evaluating the efficacy of the extracortical bone-bridging and ingrowth fixation technique in providing long-term stable stem fixation concluded that the extra-cortical bone bridging and ingrowth might contribute to extended longevity of endoprosthesis and reduce prevalence of stem-loosening^1^. The formation of EBBI may be related to the technical aspects of preparing and applying the EBBI to the bone-prosthesis junction. In our centre, multiple bone sleeves measuring about 2-5mm are prepared with a vertical length of about 5cm. It is then arranged in an overlapping manner around the bone-prosthesis junction and anchored with absorbable suture. We performed this retrospective study on the formation of EBBI at bone-prosthesis junction and the effect of factors such as age, sex, pathology, site of pathology, and chemotherapy treatment. Our results show that the mean percentage of length and mean thickness of EBBI is comparable to other studies. It is sufficient to increase the endoprosthesis's stability, thus reducing the incidence of aseptic loosening^1,10^. Overall in our study, we observed that the thickness and length of EBBI were superior in young patients, giant cell tumour that did not receive chemotherapy and distal femur that have a bulk of muscle for revascularisation proximal tibia. However, the final multivariable statistical analysis showed no significant difference. One interesting correlation was that the EBBI thickness significantly correlated with EBBI Length (p<0.001). We conclude that, for each 1mm increase in EBBI thickness, on average, the length will increase by 0.06%, as illustrated in Fig. 4. In a study by Fukuroku *et al*^9^, the mean bone ingrowth into porous space was 30.8%. The number was not far different from this study; in this study percentage of bone growth at the porous area was 33.47%. This implies that our technical aspect of producing stable EBBI is good and producible across a large group. Fig. 1 and Fig. 2 show a series of two cases managed with endoprosthesis and their outcome.

Virolainen *et al* showed in an animal model that doxorubicin, cisplatin, and ifosfamide chemotherapy has a significant negative effect on new bone formation. This is seen in reduced callus size and ultimate strength extracortical fixation being affected by the use of chemotherapy^11^. Subsequent researchers found chemotherapy temporarily adversely affected bone growth at the bone-implant interface; however, they observed no significant differences between the two groups after one year^12^. A retrospective study specifically evaluated the effect of chemotherapy on radiological evidence of loosening concluded significant changes at three years^13^.

The need for random control trial and many participants, especially with multiple variables, is essential to settle this issue. Recently, a group from Hong Kong evaluated the role of vascularised extracortical bone bridge interface using the proximal segment of bone over margin to wrap the prosthesis with a mean follow-up of 4.27 years. In their series, 27.8% showed early loosening in the non-bone graft group. The VBG group fared significantly better at six months and 12 months post-operatively than the non-VBG group in terms of radiological scoring. Age, sex, site, and chemotherapy exposure showed no significant effect in bone junction interface formation^14^.

This study presents some limitations. There were many dropouts, mainly due to the transfer of follow-ups to other tertiary centers. Other limitations included the absence of radiological image during follow-up at 24 months among participants in the earlier phase of the study. A further prospective study with a large sample size may overcome this limitation and produce more significant results.

## Conclusion

Based on our study results, there was no factor that significantly contributed to the formation and incorporation of EBBI. However, the thickness correlated with the length of EBBI formation.
